# The Prevalence of Generalized Anxiety Disorder Among Health Care Workers During the COVID-19 Pandemic: A Systematic Review and Meta-Analysis

**DOI:** 10.3389/fpsyt.2021.658846

**Published:** 2021-05-31

**Authors:** Amir Adibi, Mohamad Golitaleb, Iman Farrahi-Ashtiani, Davoud Pirani, Kosar Yousefi, Yousef Jamshidbeigi, Ali Sahebi

**Affiliations:** ^1^Department of Child and Adolescent Psychiatry, Ilam University of Medical Sciences, Ilam, Iran; ^2^Department of Nursing, School of Nursing, Arak University of Medical Sciences, Arak, Iran; ^3^Department of Health in Disasters and Emergencies, School of Public Health and Safety, Shahid Beheshti University of Medical Sciences, Tehran, Iran; ^4^Non-Communicable Diseases Research Center, Ilam University of Medical Sciences, Ilam, Iran; ^5^Clinical Research Development Unit, Shahid Mostafa Khomeini Hospital, Ilam University of Medical Sciences, Ilam, Iran; ^6^Department of Anesthesia, School of Paramedical, Ilam University of Medical Sciences, Ilam, Iran

**Keywords:** healthcare worker, COVID-19, generalized anxiety disorder, anxiety disorders, health care providers

## Abstract

**Introduction:** Health care workers, due to be involved in caring for COVID-19 patients may experience various psychological problems including anxiety disorders. This study aimed to investigate the prevalence of Generalized Anxiety Disorder (GAD) among health care workers during the COVID-19 pandemic by systematic review and meta-analysis.

**Methods:** The PRISMA guideline was used for conducting this study. Related keywords were searched in credited resources including ISC, Magiran, PubMed, Scopus, Web of Science, Cochrane, ProQuest, Science Direct, Google Scholar, and Embase to find the articles published on the prevalence of GAD among health care workers during the COVID-19 pandemic from the first of January to the end of June 2020. Meta-analysis was conducted by the random effects model.

**Results:** In this study, 553 articles were initially identified, from which 19 studies were finally included in the meta-analysis. The results showed that the prevalence of GAD in health care workers based on the GAD-7 and GAD-2 instruments were 32.04% (95% CI: 26.89–37.19, *I*^2^ = 98.2%, *p* < 0.001) and 22.62% (95% CI: 9.01–36.24, *I*^2^ = 97.7%, *p* < 0.001). The overall prevalence of GAD was obtained 30.5% (95% CI: 25.58–35.42, *I*^2^ = 98.4%, *p* < 0.001).

**Conclusion:** This study showed a relatively high GAD prevalence, as one of the fundamental psychological problems, among health care workers during the COVID-19 pandemic. Therefore, health system managers should implement preventive strategies to protect health staff from contracting the virus and monitor them for psychological problems and provide them with supportive measures if necessary.

## Introduction

COVID-19 was first reported from Wuhan, China, and then spread across the world. The outbreaks of infectious diseases such as COVID-19 are associated with increased psychological disorders and consequences (1–3). Factors such as unpredictability, uncertainty about disease control, and life-threatening severe risks have been associated with stress following the COVID-19 pandemic. On the other hand, mental disorders such as anxiety and depression are also common (4–6). So, in addition to health and economic consequences, COVID-19 also has negative impacts on mental health. Similar to other COVID-19 patients, health care workers (HCWs) are also vulnerable to emotional and mental health problems and adverse psychological consequences (7, 8) because of high workload, the shortage of Personal Protective Equipment (PPE), negative media news, the lack of support by authorities, and finally the high risk of contracting the COVID-19 infection (9). Therefore, the HCWs involved in caring for COVID-19 patients are exposed to high levels of stress. A study in Italy showed that 19.80% of HCWs experienced severe Generalized Anxiety Disorder (GAD) (10). Among HCWs, those working in the emergency department, intensive care unit (ICU), and infectious diseases ward are at a higher risk for psychiatric problems (9). Studies have also shown that the levels of fear, depression, and anxiety are higher in the treatment than administrative staff as they are in direct contact with COVID-19 patients (11). COVID-19 psychosocial assessments include surveys addressing stressors, secondary psychosocial consequences (e.g., depression and anxiety), and vulnerability indices (e.g., physical and psychological conditions) (12). This study is the first systematic review and meta-analysis assessing GAD's prevalence among health care workers during the Covid-19. Although HCWs, like other patients, are prone to the psychological consequences of COVID-19, these adverse effects may be ignored in them. This study was designed to answer one crucial research question: (1) what is GAD's prevalence among healthcare workers during the COVID-19 pandemic? As regards GAD is the most common anxiety disorder, Assessing the prevalence of GAD among HCWs can draw attention to their psychological problems and, on the other hand, help health care managers for future planning. The purpose of selecting this study is to have a specific tool and is homogeneous. Therefore, this study aimed to investigate GAD's prevalence among HCWs during the COVID-19 pandemic by systematic review and meta-analysis.

## Methods

In the present study, the guidelines of Preferred Reporting Items for Systematic Reviews and Meta-Analyses (PRISMA) were followed (13). This review's protocol has been registered in the International Prospective Register of Systematic Reviews (PROSPERO) under the code of CRD42020204428.

### Search Strategy

The search was conducted in ISC, Magiran, PubMed, Scopus, Web of Science, Cochrane, ProQuest, Science Direct, Embase and Google Scholar resources using valid English keywords and their Persian equivalents including: “Anxiety,”“Generalized Anxiety Disorder,”“GAD,” “Anxiety Disorder,” “Mental health Disorder,” “Psychiatric Disorder,” “2019 novel coronavirus disease,” “ COVID19,” “COVID-19 pandemic,” “SARS-CoV-2 infection,” “COVID-19 virus disease,” “2019 novel coronavirus infection,” “2019-nCoV infection,” “Coronavirus disease 2019,” “2019-nCoV disease,” “COVID-19 virus infection,” “Health Personnel,” “Health worker” OR “Healthcare Provider” OR “Healthcare Worker” OR “Health care profesional” OR “Medical staff” OR “Medical worker.” Applying appropriate operators, keywords and related search fields, appropriate search strategies were selected for each database. The search was conducted to include the studies published from January first to the end of June 2020.

### A Sample Search Strategy in PubMed

[(Anxiety OR “Generalized Anxiety disorder^*^” OR GAD OR “Anxiety Disorder^*^” OR “Mental health Disorder^*^” OR “Psychiatric Disorder^*^”) AND (“2019 novel coronavirus disease” OR COVID19 OR “COVID-19 pandemic” OR “SARS-CoV-2 infection” OR “COVID-19 virus disease” OR “2019 novel coronavirus infection” OR “2019-nCoV infection” OR “Coronavirus disease 2019” OR “2019-nCoV disease” OR “COVID-19 virus infection”) AND (“Health Personnel” OR “Health Care Provider^*^” OR “Health worker^*^” OR “Healthcare Provider^*^” OR “Healthcare Worker^*^” OR “Health care professional^*^” OR “Medical staff” OR “Medical worker^*^”)].

#### Inclusion and Exclusion Criteria

In this review, all the studies reporting GAD's prevalence in either Persian or English were included. Reporting means GAD scores, anxiety level, and having an interventional design were considered exclusion criteria. Also, letters to the editors and systematic reviews were excluded.

#### Study Selection

At first, 553 primary articles retrieved from the resources were inserted into EndNote X7 reference manager software. After removing 128 duplicate studies, the titles and abstracts of 425 articles were screened, and 86 studies were selected for further evaluation. At this stage, two researchers independently studied the full texts of these 86 articles in detail, which resulted in selecting 19 studies for qualification.

#### Qualification and Data Extraction

Initially, two researchers independently assessed the selected studies' quality using the STROBE standard checklist (14). The minimum and maximum scores on this checklist were 0 and 44, respectively, and those studies that attained at least 16 scores (15) were selected to be included in the meta-analysis. For data extraction, the same two researchers independently extracted the required data (first authors' names, study location, sample size, GAD prevalence, the number of males and females, and the utilized tools) from the final studies using a checklist prepared by the research team (Table 1).

**Table 1:** The characteristics of the extracted articles investigating the prevalence of generalized anxiety disorder among health care workers during the COVID-19 pandemic.

**References**	**Place**	**Total sample size**	**Prevalence of GAD**	**Instrument**	**Male**	**Female**
Yang et al. (16)	South korea	65	32.3%	GAD−7	34	31
Fu et al. (17)	China	454	35.2%	GAD−7	–	–
Que et al. (18)	China	2,285	46.04%	GAD−7	707	1,578
Civantos et al. (19)	USA	349	47.9%	GAD−7	212	137
Huang and Zhao (20)	China	2,250	35.6%	GAD−7	–	–
Lai et al. (21)	China	1,257	44.6%	GAD−7	293	964
Zhu et al. (22)	China	5,062	24.1%	GAD−7	758	4,304
Zhang et al. (23)	China	927	13%	GAD−2	249	678
Temsah et al. (24)	Saudi Arabia	582	15.92%	GAD−7	145	437
Motta et al. (25)	China	4,369	25.2%	GAD−7	–	4,369
Ma et al. (26)	China	34	35%	GAD−7	10	24
Zhang et al. (27)	China	1,563	44.7%	GAD−7	270	1,293
Shechter et al. (28)	USA	657 (4)^*^	33%	GAD−2	147	509
Naser et al. (29)	Jordan	1,163	28.73%	GAD−7	510	653
Chen et al. (30)	Ecuador	252	28.2%	GAD−7	87	165
Ni et al. (31)	China	214	22%	GAD−2	67	147
Gupta et al. (32)	India	123	12.20%	GAD−7	–	–
Apisarnthanarak et al. (33)	Thailand	160	18.02%	GAD−7	65	95
Tu et al. (34)	China	100	40%	GAD−7	–	100

* *Gender unknown*.

#### Statistical Analysis

The random-effects model was used for meta-analysis, and the *I*^2^ index was exploited to check heterogeneity among the studies. The *I*^2^ index values of <25%, 25 to 75%, and 75% or more indicate low, medium, and high heterogeneity, respectively (35). Any association between the prevalence of GAD and sample size was investigated by meta-regression. In this study, the Egger's and Begg's tests were used to assess publication bias. The data were analyzed by STATA (version 14) software.

## Results

**Figure 1:**
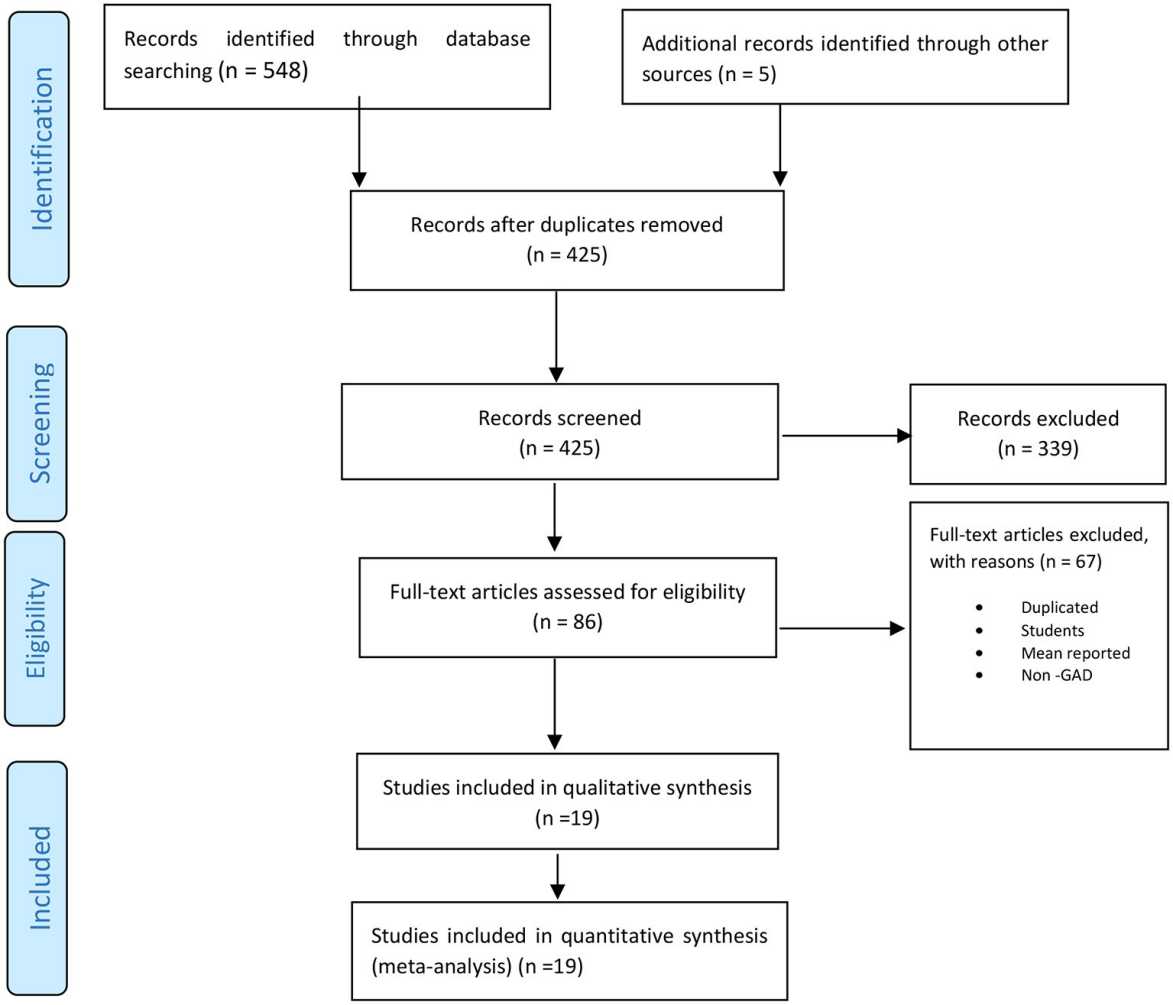
Flowchart of the selection of studies based on PRISMA.

Based on a comprehensive search, 553 studies were initially extracted, and after removing duplicates, 425 studies were screened, from which 86 were selected for reviewing full texts. Finally, 19 studies were chosen for quality assessment, and all of them were included in the meta-analysis. The study selection process has been shown in Figure 1.

In this study, 21,866 HCWs were evaluated, of whom 3,550 were men, and 15,484 were women (the gender of 4 participants remained unknown). All the studies had used one of the GAD-7 and GAD-2 tools to determine the prevalence of GAD among HCWs. The GAD-7 has seven items, and each item is scored from 0 to 3 with a final score ranging from 0 to 21. The final scores of 0–4, 5–9, 10–14, and 15–21 indicate no (or minimal), mild, moderate, and severe GAD, respectively (36). The GAD-2 instrument also has two items, each with a score ranging between 0 and 6. A score higher than 3 indicates the presence of GAD (37).

**Figure 2:**
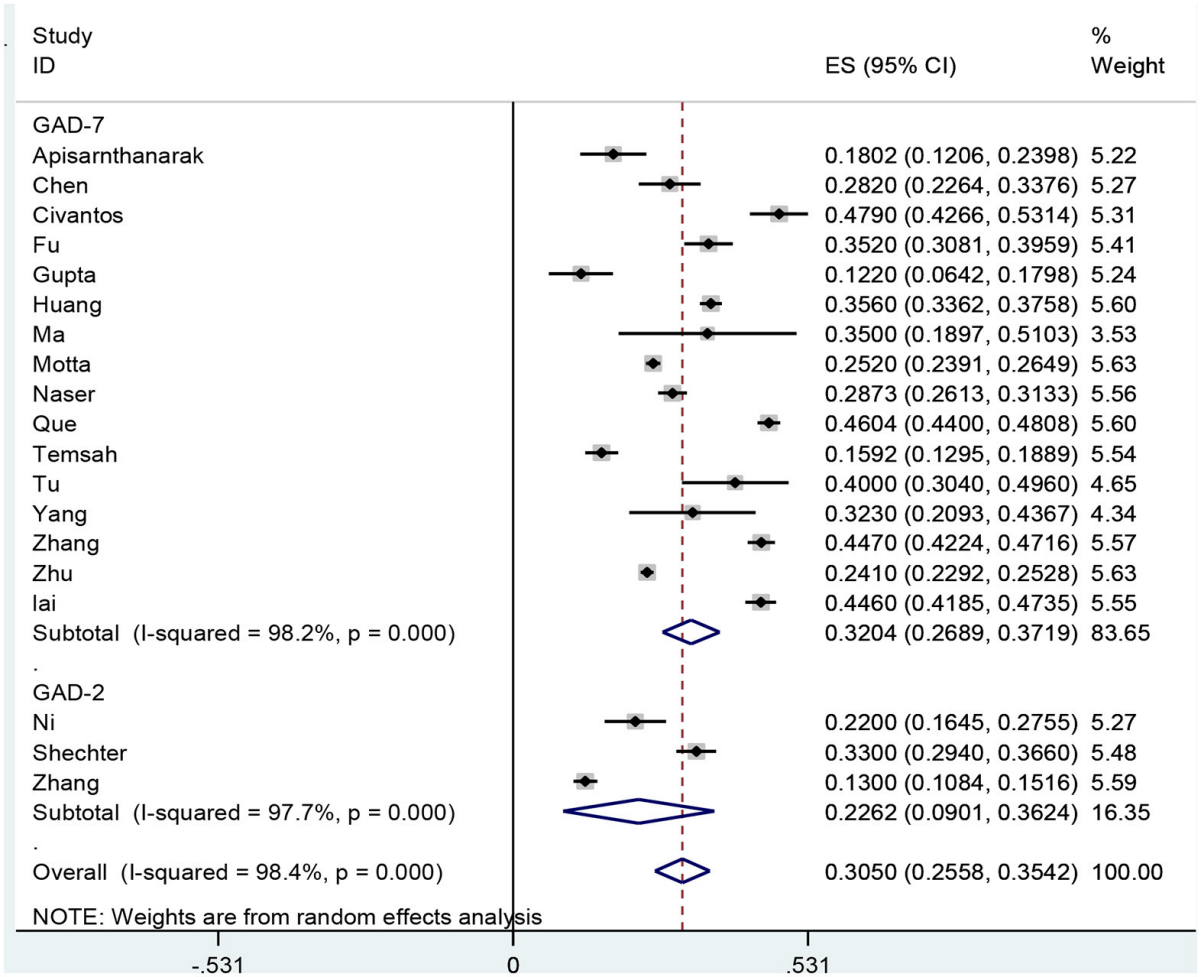
The forest plot of GAD prevalence among health care workers during the COVID-19 pandemic.

**Figure 3:**
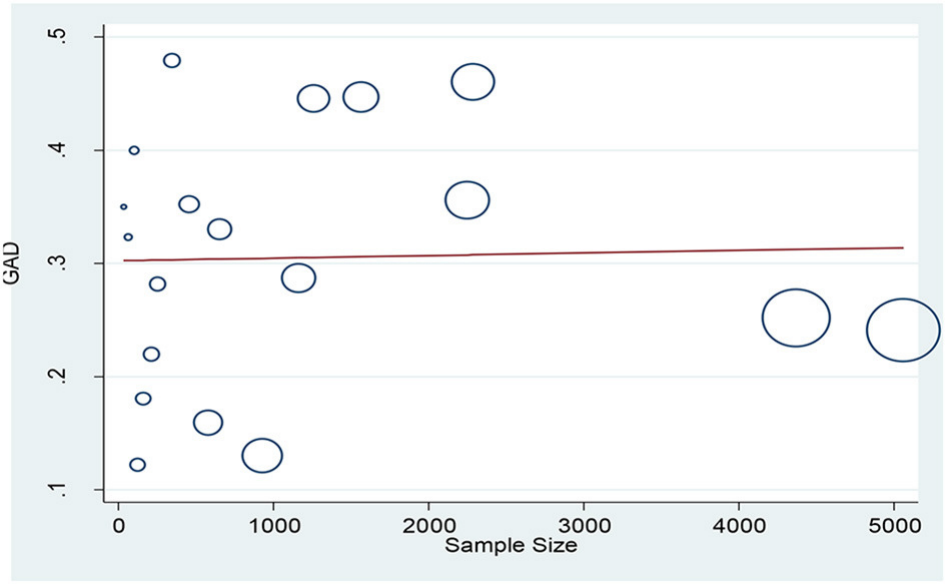
The association between GAD prevalence among health care workers and sample size based on meta-regression analysis.

**Figure 4:**
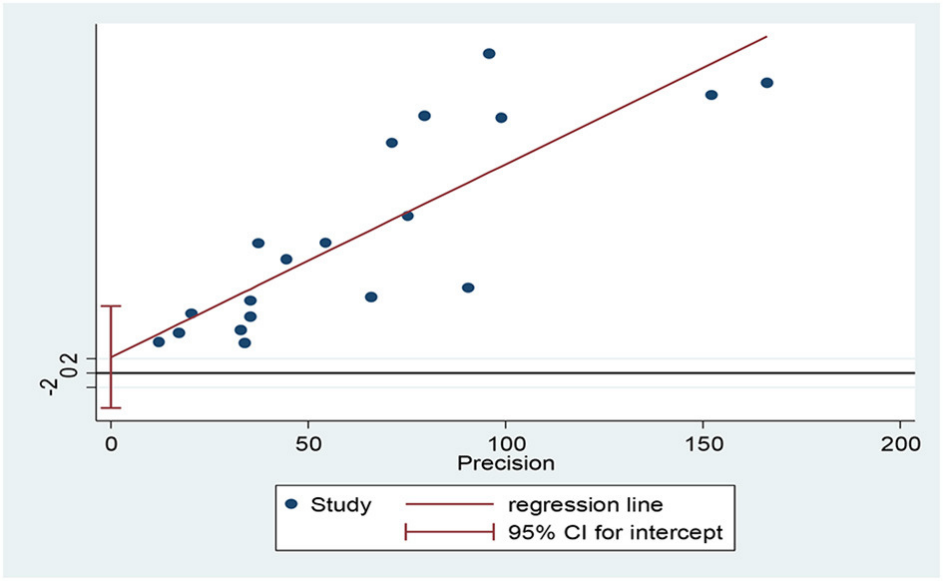
Publication bias based on the egger test.

According to the results, the prevalence of GAD among HCWs were obtained 32.04% (95% CI: 26.89–38.19, *I*^2^ = 98.2%, *p* < 0.001) and 22.62% (95% CI: 9.01–36.24, *I*^2^ = 97.7%, *p* < 0.001) based on the GAD-7 and GAD-2 instruments, respectively. The overall prevalence of GAD was calculated as 30.5% (95% CI: 25.58–35.42, *I*^2^ = 98.4%, *p* < 0.001; Figure 2). Meta-regression analysis showed that with increasing sample size, GAD's prevalence also increased (Figure 3). According to Egger's (*P* = 0.519) and the Begg's (*P* = 0.972) tests, publication bias was not considerable in the present study (Figure 4).

## Discussion

Based on the present review results, GAD's prevalence among HCWs during the COVID-19 pandemic was 32.04 and 22.62%, according to the GAD-7 and GAD-2 instruments, respectively. Also, the overall prevalence of GAD among HCWs was obtained as 30.5%. In a meta-analysis study by Salari et al. who examined the prevalence of anxiety, stress, and depression in the general population during the COVID-19 pandemic, the prevalence of anxiety, stress, and depression were reported as 31.9, 29.6, and 33.7%, respectively (38). The results of the present and the studies mentioned above indicate that HCWs, similar to the general population, experience anxiety during the COVID-19 pandemic. In another study, Salzar De Pablo et al. reported the prevalence of fear, insomnia, occupational burnout, and post-traumatic stress disorder (PTSD) among HCWs during the COVID-19 pandemic as 43.7, 37.9, 34.4, and 20.7%, respectively (39). Based on this observation, one can conclude that HCWs, in addition to GAD, may also experience many other psychological problems during the pandemic. Anxiety has been shown to predispose these individuals to a variety of mental disorders. A cross-sectional study in 2018 reported a prevalence of 28.6% for anxiety in the hospital personnel working in the emergency department (40). Another cross-sectional study in 2019 reported a prevalence of 33.8% for GAD (based on the GAD-7 survey) in medical students (41). Considering the results of these studies, it can be concluded that the epidemics of infectious diseases such as the COVID-19 can increase the incidence of anxiety among HCWs.

Studies have shown that the COVID-19 is an independent risk factor for stress among HCWs. Furthermore, factors such as age, gender, workplace, and inadequate psychosocial support have been associated with depression and anxiety among HCWs (9). During the Ebola epidemic, HCWs who had direct contact with the patients experienced more mental health disorders. Therefore, it is essential to include mental health experts in the context of emergency response programs to emerging infectious diseases (42). On the other hand, HCWs should be prepared for the potential psychological outcomes of infectious diseases outbreaks, and managers should support the personnel who are at the highest risk of contracting the infection and those most involved in caring for patients (43). The present study results showed HCWs are highly exposed to anxiety disorders, especially GAD, during the COVID-19 pandemic, and special attention should be paid to their mental health. Negligence in the proper management of HCWs' psychological problems may have dire consequences as the poor mental health of nurses affects their performance and the quality of the care provided to patients (44, 45). In addition to the risk of being infected with COVID-19 disease, HCWs are also at the risk of developing anxiety disorders. Because the vulnerability to and the risk factors of psychological disorders may differ in individuals, it is recommended to investigate anxiety disorders' risk factors among HCWs in future studies.

## Conclusion

The present study results showed that HCWs, in addition to COVID-19, are exposed to its various psychological consequences. Since HCWs are at the frontline of the battle with the COVID-19 disease and in direct contact with the patients, health care managers, and considering preventive measures to protect HCWs against the COVID-19 disease, should also pay attention to their psychological health and take necessary supportive measures if necessary.

The findings of this study can be used as a database for psychiatrists and health managers. This study's clinical implications include creating a sensitivity at all health management levels to prevent, timely diagnose, and manage the fear of anxiety and treat GAD in vulnerable healthcare workers by implementing appropriate interventions and programs. The interventions that can be considered to reduce GAD include: Creating a suitable environment for effective communication, limiting shift change times, providing a place for resting, providing extensive access to protective equipment and implementing strict rules on their use and management, and providing specialized training about the treatment process of COVID-19 patients. Providing timely and appropriate support, including mental health professionals use for consulting with healthcare workers and education through media and multimedia programs, lectures, group counseling, individual counseling, online platforms, and implementing mental health phone lines, can help.

## Limitations

One of the limitations of this study was that the analyzed studies did not report GAD's prevalence in individual genders. In some studies, that used the GAD-7 tool, the severity of GAD had been reported instead of its overall prevalence, which in these cases and considering a cut-off value of ≥5, the research team used the weighted average percentage to report the overall prevalence of GAD. In some of the studies, however, it was not possible to calculate the weighted average percentage. A high heterogeneity among the studies, which may reflect large differences in their sample sizes, was another limitation.

## Data Availability Statement

The original contributions presented in the study are included in the article/supplementary material, further inquiries can be directed to the corresponding author/s.

## Author Contributions

AS, KY, AA, and MG designed the review, developed the inclusion criteria, screened titles and abstracts, appraised the quality of included papers, and drafted the manuscript. AS, AA, DP, YJ, IF-A, and MG reviewed the study protocol and inclusion criteria and provided substantial input to the manuscript. AS, IF-A, YJ, DP, and KY reviewed the study protocol. AS and AA read and screened articles for inclusion. All authors critically reviewed drafts and approved the final manuscript.

## Conflict of Interest

The authors declare that the research was conducted in the absence of any commercial or financial relationships that could be construed as a potential conflict of interest.
